# The function and regulation network mechanism of circRNA in liver diseases

**DOI:** 10.1186/s12935-022-02559-1

**Published:** 2022-03-31

**Authors:** Panpan Wang, Yunhuan Zhang, Lugang Deng, Zhi Qu, Peisen Guo, Limin Liu, Zengli Yu, Peixi Wang, Nan Liu

**Affiliations:** 1 College of Public Health, Zhengzhou University, Zhengzhou, 540001 People’s Republic of China; 2 Institute of Chronic Disease Risks Assessment, School of Nursing and Health, Henan University, Kaifeng, 475004 People’s Republic of China; 3 South China Hospital, Health Science Center, Shenzhen University, Shenzhen, 518116 People’s Republic of China

**Keywords:** circRNA, Liver diseases, Hepatocellular carcinoma (HCC), Biomarker, miRNA sponge

## Abstract

Circular RNA (circRNA), a new type of endogenous non-coding RNA, is abundantly present in eukaryotic cells, and characterized as stable high conservation and tissue specific expression. It has been generated increasing attention because of their close association with the progress of diseases. The liver is the vital organ of humans, while it is prone to acute and chronic diseases due to the influence of multiple pathogenic factors. Moreover, hepatocellular carcinoma (HCC) is the one of most common cancer and the leading cause of cancer death worldwide. Overwhelming evidences indicate that some circRNAs are differentially expressed in liver diseases, such as, HCC, chronic hepatitis B, hepatic steatosis and hepatoblastoma tissues, etc. Additionally, these circRNAs are related to proliferation, invasion, migration, angiogenesis, apoptosis, and metastasis of cell in liver diseases and act as oncogenic agents or suppressors, and linked to clinical manifestations. In this review, we briefly summarize the biogenesis, characterization and biological functions, recent detection and identification technologies of circRNA, and regulation network mechanism of circRNA in liver diseases, and discuss their potential values as biomarkers or therapeutic targets for liver diseases, especially on HCC.

## Introduction

CircRNA, as a type of endogenous non-coding RNA (ncRNA), was first found in the virus in 1976. But it was considered as one of the non-function by-products from splicing errors in a long time [1–3], thus, it was thought unlikely to play important role in biological processes. With the development of high-throughput sequencing technology and bioinformatics, circRNA has been found to be abundantly present in eukaryotic cells [4]. It is an abnormally stable RNA molecule by covalently closed loop structure, therefore the expression of circRNA is higher than linear mRNA, miRNA, and other types of RNA in whole blood, plasma, and platelets. Furthermore, circRNA exhibits longer half-time and more detectable than that of linear RNA by resisting to RNA exonuclease or RNase R. Hence, it is not surprising that circRNA could be ideal biomarker for disease [5, 6]. Besides, circRNA is involved in various physiological and pathological pathways by regulating gene expression and protein activity [7]. Increasing evidences indicate circRNAs have a strong association with diseases including systemic lupus erythematosus [8], disease of the central nervous system [9, 10], diabetes [11], cancers [12] and liver diseases [13], etc.

Liver disease accounts for significant morbidity, economic burden and costs [14], and leads to approximately 2 million deaths per year worldwide, of which one million deaths were from cirrhosis complications, and the other 1 million deaths were from viral hepatitis and hepatocellular carcinoma (HCC) [15]. Worldwide, liver cancer is the most common hepatic malignancies, ranking fifth in incidence rates and third in cause of cancer-linked deaths [16]. In addition, chronic infections with hepatitis B (HBV) were responsible for 33% of liver cancer death, alcohol for 30%, HCV for 21%, and other causes for 16% deaths in 2015 [17]. Unsatisfactorily, there is a lack of more effective diagnostic and prognostic markers besides α-fetoprotein (AFP) [18, 19]. More than 75% of patients with liver disease are often diagnosed in terminal-stage of HCC and the overall ratio of mortality to incidence is 0.95 [20, 21]. Moreover, although the 5-year overall survival rate is as high as 50%, more than 70% patients have a recurrence [22], which seriously affects the prognosis of HCC. Therefore, it is very urgent to elucidate the pathogenesis of HCC and to find valuable targets for diagnosis and treatment.

Growing evidences indicate that several dysregulated circRNAs are involved in HCC and play a vital role in many biological processes [23]. In this review, we briefly described the biogenesis, characterization, biological functions, detection and validation technologies of circRNAs. In addition, we introduced the network regulation mechanism of circRNAs in liver diseases. It not only shows that circRNAs play critical roles in the progression of liver diseases but also that they may be employed as new diagnostic and prognostic biomarkers.

## Biogenesis and characterization of circRNA

**Fig. 1:**
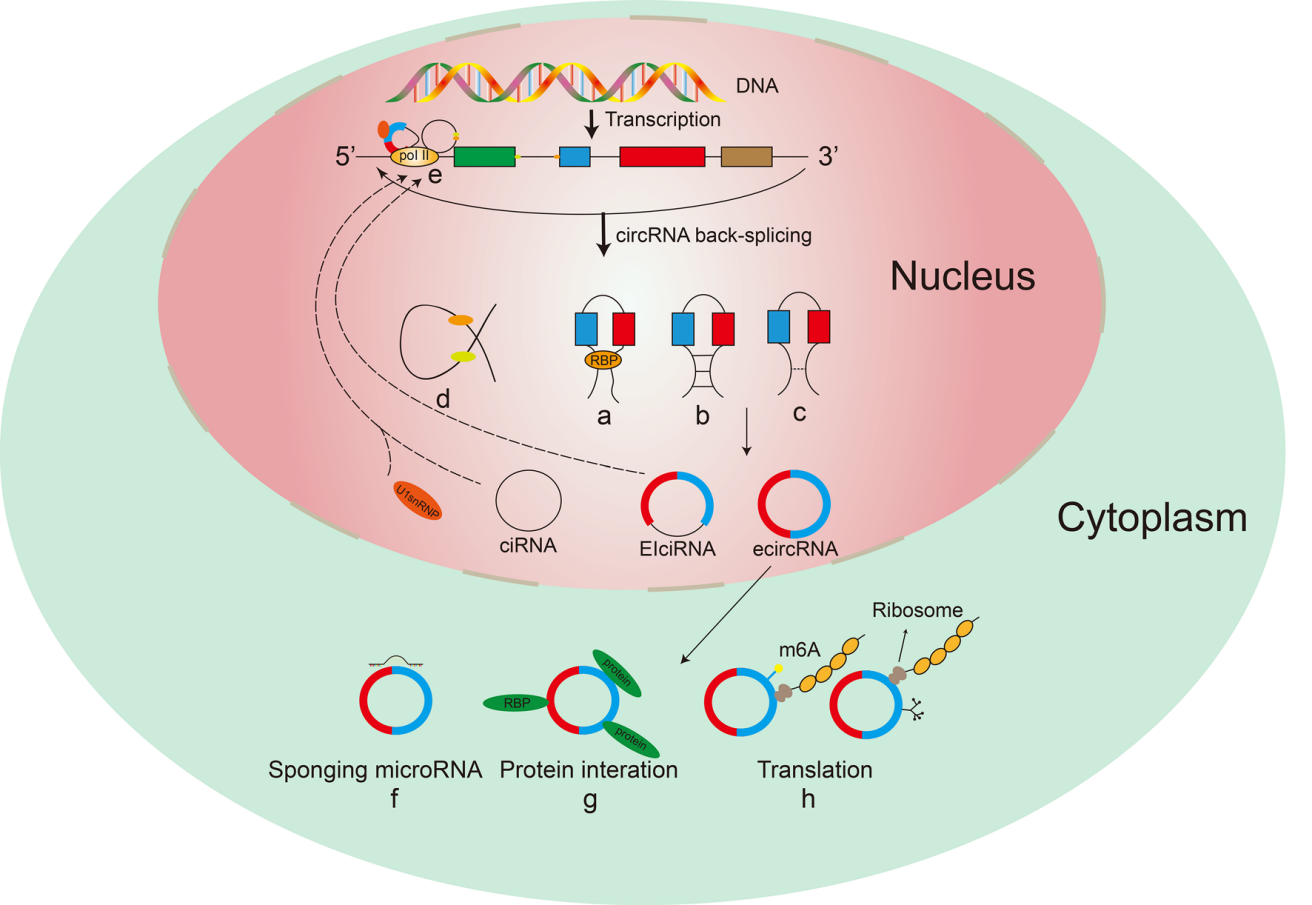
Biogenesis and function of circRNA. **a** RBP-driven circularization; **b** Intron pairing-driven circularization; **c** Lariat-driven circularization; **d** Lariat introns-driven circularization; **e** Gene transcription regulation; **f** sponging miRNA; **g** protein interaction; **h** transcription regulation

Unlike linear RNA, circRNA forms as covalently closed loop structures with neither poly-adenylated tail nor 5′–3′ polarity via back-splicing. It can be simply classified into three categories according to their composition and sequence: exonic circRNA (ecircRNA), circular intronic RNA (ciRNA) and exon–intron circRNA (EIciRNA) (Fig. 1). Though the circularization mechanisms of circRNA have not been clarified. There are four mechanisms are suggested to explain the biogenesis of circRNA: (1) Lariat-driven circularization (exon skipping), during the splicing of pre-mRNA, the 3′ splice acceptor upstream of the exon interconnects with the 5′ splice donor downstream to produce a lasso structure containing the exon and intron, if the intron is removed, ecircRNAs or EIciRNAs are generated [24]. (2) Intron pairing-driven circularization, when there are opposite complementary sequences in the flanking introns of circRNA, upstream introns and downstream introns complement each other based on reverse repeat and complementary sequences, and introns are removed or retained to form ecircRNAs or EIcirRNAs, respectively [25]. (3) RNA-binding protein (RBP)-driven circularization, in this model, RBP can promote the interaction between upstream introns and downstream introns, resulting in the production of ecircRNAs or EIciRNA [26]. (4) Lariat introns-driven circularization, the formation of the lasson introns-driven circularization mainly refers to the combination of the 7-nt GU rich element near the 5 splicing point and the 11-nt C-rich element near the 3′ branching point to escape the degradation of the debranching enzyme and form a stable ciRNA [27].

CircRNA has the following specific characteristics. (1) In recent years, circRNA has been found to be abundant in almost all species, e. g., human [28], mouse [28], zebrafish [29] and plants [30], etc. It was reported that there are 1950 species of circRNAs in human more than that of nematode and mouse [31]. Additionally, circRNA can be detected in human saliva, blood, urine, and exosomes, which can be employed as novel and potential biomarkers [32]. (2) CircRNA is highly stable in eukaryotes and covalently closed, which can protect them from exonuclease-mediated degradation. (3) The expression of circRNA varied with time, space and tissue type. For instance, some neuro-associated circRNAs were found to increase with age in *Drosophila* [33]. There are also obvious differences in spatial expression, such as most ecircRNAs are found in the cytoplasm, and ciRNA is present in the nucleus. Besides, some circRNAs originate from human and mouse which are tissue-specific and mostly expressed in the brain [34]. (4) It is highly conserved in different species, Jeck et al. [35] found that there are 69 circRNAs in mouse testes are precisely homologous to human fibroblasts. These characteristics indicate that circRNA plays an important role in the occurrence and development of the disease and serves as potential biomarkers in bodily fluid for disease diagnosis.

## Biological functions of circRNA

CircRNAs were found to have many potential biological functions on the basis of its characteristics, such as the following (Fig. 1): (1) Nuclear retained circRNAs can modulate transcription and splicing. Li [36] found that it participated in transcription regulation by interacting with U1 small nuclear ribonucleoproteins (U1snRNPs), the EIciRNA-U1 snRNP complexes bind to Pol II at the promoters of its parental genes to enhance gene expression. CircSEP3, as a nuclear retained circRNA roots in exon 6 of SEPALLATA3 in arabidopsis, was reported to regulate the splicing of its linear counterpart [37]. (2) CircRNA can act as miRNA sponge. The structure of circRNAs displayed numerous miRNA binding sites which assist the interactions with miRNAs [38]. CiRS-7 has been reported to contained 70 conserved binding sites of miR-7, and then it could increase expression level of miR-7 target by sponging miR-7 [39]. (3) circRNAs can perform its function by interacting with proteins, such as serving as protein sponge, protein scaffolding and protein recruitment, etc. [10, 40]. For example, circFOXO3 can promote cardiac senescence act as a scaffold to connect with the anti-senescent protein ID-1 and the transcription factor E2F1 as well as the anti-stress proteins FAK and hypoxia-inducible factor-1α (HIF1A) [41]. Besides, circRNA derived from human antigen R (HuR), an RNA binding protein (RBP) with AU-rich elements. And circHuR could directly interacted with CCHC-type zinc finger nucleic acid binding protein and inhibited its binding to HuR promoter, then reducing the expression level of HuR and restricting the progression of gastric cancer [42]. (4) circRNA can be translatable. Although most of the circRNAs haven’t been associated with ribosomes for translation, a small part of endogenous circRNAs are translatable, such as the human circHO which was found to encode protein and control the myoblast proliferation in human and murine and human [43]. Besides, recent studies have shown that N6-methyladenosine (m6A) modification can regulate the translation and degradation of circRNA, thus affecting the occurrence and development of tumors [44].

## Detection and validation methods for circRNA

**Fig. 2:**
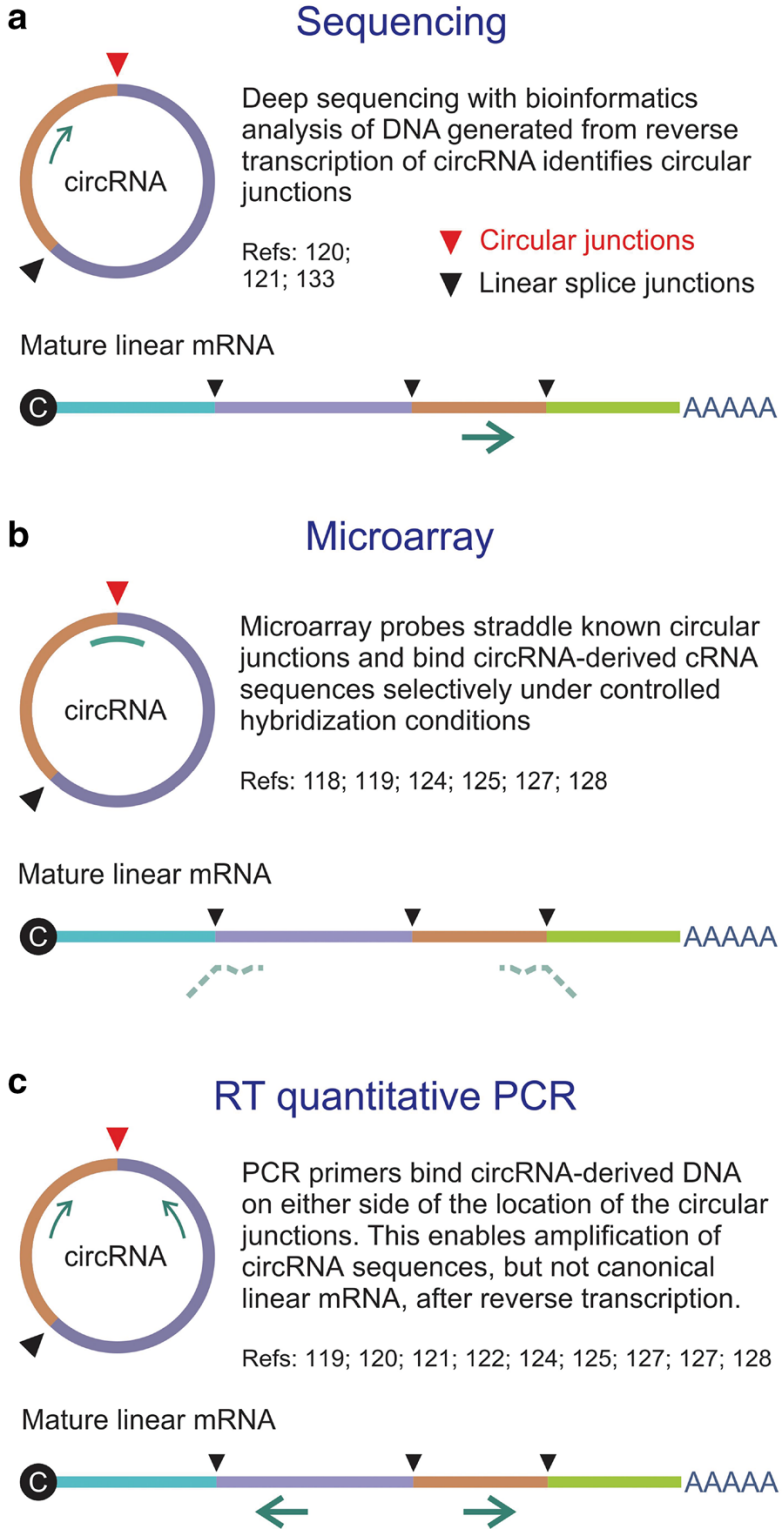
Methods for assay of circRNA. **a** direct sequencing of a reverse transcribed circRNA typically employs a primer that is complementary to an exon. The sequencing reaction does not discriminate between linear RNA and circRNA, but bioinformatics analysis allows identification of circular junctions (red arrowhead); **b** Microarrays make use of probes that straddle unique and defined circularization junctions. Typically random primers are used to generate labeled cRNAs from circRNA templates. Probes span the circular junctions and hybridize to the cRNAs with higher Tms than the partly complementary sequences of linear mature RNA; **c** Reverse transcriptase (RT) quantitative PCR entails specific amplification of sequences derived from circRNAs by using primers that flank the circular junctions. The configuration of the amplifying primers is such that mature linear mRNA is not amplified. Reprinted with permission from Abdullah et al. [46] Copyright© 2021 Dovepress

Technologies and methods to analyze and identify circRNA have greatly and considerably improved with the developments of sequencing technology and biochip technology; however, error and bias that influence accuracy are yet to be completely eliminated [45]. With advances in sequencing technology including RNA sequencing (RNA-Seq), next generation sequencing (NGS), microarrays etc., and bioinformatics tools consisting of circRNA annotations, circRNA identification and network analysis of ceRNA, the types and biological functions of circRNAs are expanding, such as biomarkers for diseases [46]. Common detection and verification methods of circRNA are introduced in this section (Fig. 2).

### Detection methods for circRNA

At present, RNA-Seq and microarrays are widely applied for circRNA detection. RNA-seq is primarily used for the discovery of novel circRNAs. The head-to-tail junctions are the only direct evidence for detecting circRNAs [47]. Generally, RNaseR is used to treat the total RNA to demonstrate the stability of circRNAs by eliminating linear RNAs, but not circRNAs. Then, microarray probes hybridized to the fluorescently labeled cRNA, which is from circRNA and tarted the head-to-tail junctions. As an extensive and alternative genome-wide assay, circRNA-specific microarray has been demonstrated great specificity and sensitivity compared to NGS; e. g., the related products of Arraystar (https://www.arraystar.com/) and Cofactor Genomics (https://cofactorgenomics.com/) [46]. In addition, circRNA-associated bioinformatics tools including circRNA_finder, find_circ, CIRCexplorer, CIRI and MapSplice, etc., can identify circRNA mainly based on the presence of backsplice junction-spanning sequencing reads. Recently, more and more circRNAs have been discovered in liver diseases through the above-mentioned technologies and have been proved to be differentially expressed by bioinformatics tools [48]. For example, Guan et al. [49] and Yu et al. [50] detected 1245 (756 up-regulation and 489 down-regulation) circRNAs and 257 differedtial expression circRNAs (DECs) (213 up-regulation and 44 down-regulation) from three pairs of HCC tissue and the microarray dataset GSE78520 by using microarray, respectively. RNA-seq can be employed for detection of circRNAs by RNA fragmentation, capture, sequencing, and subsequent computational analysis. Hu et al. [51] detected 72, 277 known circRNAs and identified 220 DECs by RNA-seq in 30 primary HCC tissues, including 15 HCC tissues with pulmonary metastasis after curative resection and 15 normal tissues without pulmonary metastasis. Moreover, further researches for DECs are contributed to finding the diagnostic markers and therapeutic targets for liver diseases. Among all of the 1245 DECs, Guan et al. [52] also reported that hsa_circ_0016788 had a highly potential diagnostic value and might be a promising biomarker of HCC. Qiao et al. [53] detected that the expression of hsa_circ_0003998 which was the most significantly upregulated among the 22 DECs (14 up-regulated and 8 down-regulated) in HCC tissues by RNA-seq. In addition, hsa_circ_0003998 was related to serum AFP level and clinicopathological factors, which suggesting it would be an early diagnostic biomarker of HCC.

### Validation methods for circRNA

Based on the development of second-generation sequencing and bioinformatics analysis, it is possible to sequence hundreds of millions of short reads. DECs are required to be experimentally verified in the later stage [54]. Standard PCR-based methods including reverse transcription quantitative PCR (RT-qPCR), droplet digital PCR (dd PCR) and northern blot are considered as most widely employed methods for validation and more detailed quantitation of circRNAs [55–57]. Besides, Fluorescence in-situ hybridization is used to locate the distribution and abundance circRNA for further study of subsequent functions [58]. At present, a number of online databases have been established to analyze the information, regulatory network and the role of circRNA in diseases and other physiological processes, such as circBase, CIRCpedia, CircInteractome and Circ2Traits [59–62]. With increasing and continuous improvement of circRNA identification techniques and databases, the role of circRNAs will be more fully clarified.

## CircRNA in liver diseases

Liver is a critical organ of the human body, it not only has many biological functions such as controlling metabolism, maintaining energy balance and detoxifying, etc. While, it has a powerful ability to regenerate and repair after injury [63, 64]. However, various pathogenic factors such as drug, alcohol, and virus damage the liver, resulting in acute or chronic liver disease and even HCC [65]. Some evidences suggest that circRNA is involved in regulating liver homeostasis and disease. The latest report showed [66] that 668 circRNAs are specifically expressed in liver tissue of six adult and fetal normal tissues. Li et al. [67] detected that 2412 circRNAs, including 159 circRNAs deriving from 116 host linear transcripts differentially expressed in priming phase of rat liver regeneration and clarified that circRNA abundance is associated with proliferation. Therefore, it suggests circRNA is highly expressed in liver and closely related to liver diseases.

### CircRNA in HCC

**Fig. 3:**
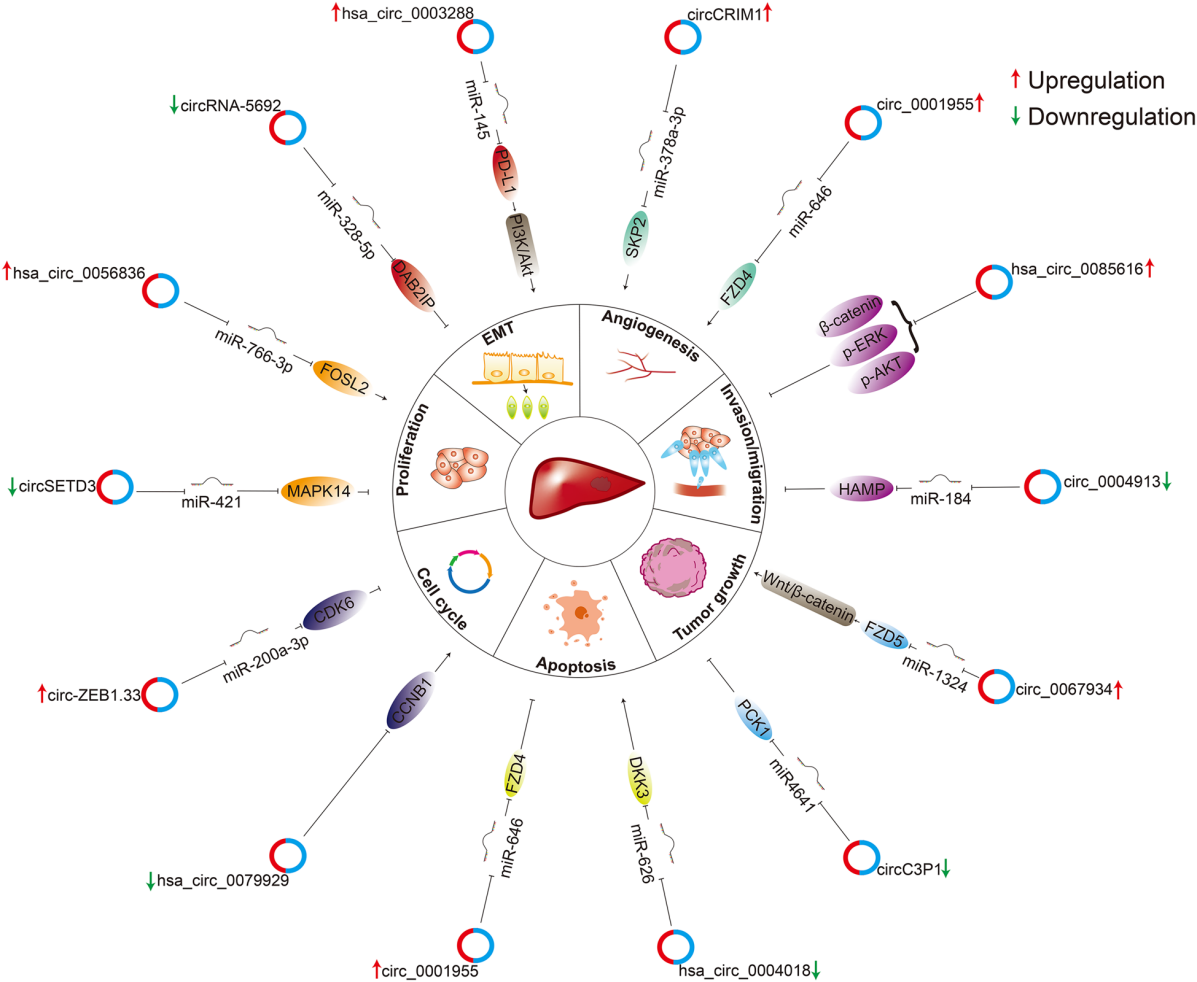
Roles and regulation network mechanism of HCC-related circRNAs. The diagram shows oncogenic agent and suppressor circRNAs in HCC and their influence on the biological processes, including proliferation, EMT, angiogenesis, invasion/migration, tumor growth, cell cycle and apoptosis. Oncogenic agent and suppressor circRNAs indicated in red and green arrow, respectively

Recently, compelling evidences already have proved that dysregulation of circRNA is associated with the development of HCC. CircRNAs is involved in proliferation, migration, invasion and apoptosis biological behaviors in HCC patients. According to the role of circRNAs, they can be divided into oncogenic agents and suppressors. Besides, circRNAs could be detected in tissue and body fluid, they are increasingly being used as potential diagnostic and prognostic markers for HCC. Here, we mainly focus on the regulation network mechanism of circRNA in HCC which illustrated in Fig. 3.

### CircRNA as oncogenic agent in HCC development

With development of circRNA microarray analysis and RNA-Seq detection, circRNA is highly expressed in biological samples including tissues, cell lines and serum of patients and acts as oncogenic agent in HCC. Previous studies have demonstrated that many miRNAs and target genes act as oncogenic agents in HCC, summarizing the role of circRNA in HCC will help us to better understand the pathogenesis of HCC and explore efficient and therapeutic targets. The following circRNAs can be used as oncogenic agents for HCC and are displayed in Table 1.

**Table 1:** circRNA as potential oncogenic agent in HCC

Name of circRNA	Source of sample	Expression	Function	Mechanism/pathway	Refs.
Cdr1as	Tissues Cell lines	Upregulation	Proliferation (+) Migration (+)	Cdr1as/miR-1287/Raf1	[76]
circHIPK3	Tissues Cell lines Animal	Upregulation	Proliferation (+) Migration (+) Growth (+)	circHIPK3/miR-124/AQP3	[70]
circPVT1	Tissues Cell lines Animal	Upregulation	Proliferation (+) Migration (+) Growth (+) Apoptosis (−)	circPVT1/miR-203/HOXD3 circPVT1/miR-3666/SIRT7	[68, 94]
circ_0015756	Tissues Cell lines Animal	Upregulation	Proliferation (+) Invasion (+) Migration (+) Growth (+)	circ_0015756/miR-7/FAK/Akt	[157]
circ_0091579	Tissues Cell lines	Upregulation	Proliferation (+) Invasion (+) Migration (+)	circ_0091579/microRNA-490-3p	[75]
circZNF609	Tissues Cell lines	Upregulation	Proliferation (+) Migration (+) Stemness (+)	circZNF609/miR-15a-5p, 15b-5p/GLI2/Hedgehog	[81]
hsa_circ_0003288	Tissues Cell lines	Upregulation	Migration (+) Invasion (+)	hsa_circ_0003288/miR-145/PD-L1 PI3K/Akt	[77]
has_circ_0039053	Tissues Cell lines Animal	Upregulation	Proliferation (+) Invasion (+) Growth (+)	has_circ_0039053/miR-637/USP21	[73]
circRASGRF2	Tissues Cell lines Animal	Upregulation	Proliferation (+) Migration (+) Growth (+)	circRASGRF2/miR-1224/FAK	[74]
hsa_circ_0056836	Tissues Cell lines Animal	Upregulation	Proliferation (+) Invasion (+) Migration (+) Growth (+)	hsa_circ_0056836/miR-766-3p/FOSL2	[69]
circGprc5a	Tissues Cell lines Animal	Upregulation	Proliferation ( +) Apoptosis (−) Growth (+)	circGprc5a/miR-1283/YAP1/TEAD1	[96]
circBACH1	Tissues Cell lines Animal	Upregulation	Proliferation (+) Growth (+)	CircBACH1/HuR/p27	[82]
circ-ZEB1.33	Tissues serum Cell lines	Upregulation	Proliferation (+)	circ-ZEB1.33/miR-200a-3p/CDK6	[83]
circ‐HOMER1	Tissues Cell lines	Upregulation	Proliferation (+) Invasion (+) Migration (+) Apoptosis (−)	circ‐HOMER1/miR‐1322/CXCL6	[97]
Cul2 circRNA	Tissues Cell lines Animal	Upregulation	Proliferation (+) Invasion (+) Migration (+) Growth (+)	Twist1/Cul2 circRNA/Vimentin	[87]
circCRIM1	Tissues Cell lines	Upregulation	Proliferation (+) Angiogenesis (+)	circCRIM1/miR-378a-3p/SKP2	[89]
circ_0001955	Tissues Cell lines	Upregulation	Proliferation (+) Migration (+) Angiogenesis (+) Apoptosis (−)	circ_0001955/miR-646/FZD4	[90]
circGFRA1	Tissues Cell lines	Upregulation	Proliferation (+) Migration (+) Angiogenesis (+)	circGFRA1/miR-149	[91]
circ_0067934	Tissues Cell lines	Upregulation	Growth (+) Metastasis (+)	circ_0067934/miR-1324/FZD5/Wntβ-catenin axis	[78]
circZFR	Tissues Cell lines	Upregulation	Proliferation (+) Invasion (+) Migration (+) Apoptosis (−)	circZFR/miR-3619–5p/CTNNB1/Wntβ-catenin circZFR/miR-511/AKT1/β-catenin CircZFR/MAP2K1	[79, 80]
hsa_circ_0085616	Tissues Cell lines	Upregulation	Proliferation (+) Migration (+) Invasion (+) Apoptosis (−)	β-catenin, p-ERK, and p-AKT	[84]
hsa_circ_102559	Tissues Cell lines Animal	Upregulation	Migration (+) Growth (+)	hsa_circ_102559/miR-130a-5p/ANXA2	[95]
circFAT1	Tissues Cell lines Animal	Upregulation	Invasion (+) Proliferation (+) Growth (+)	circFAT1/miR-30a-5p/REEP3	[98]
circCSPP1	Tissues Cell lines Animal	Upregulation	Proliferation (+) Invasion (+) Migration (+) Apoptosis (−) Growth (+)	circCSPP1/miR-1182/RAB15 circCSPP1/miR-577/CCNE2	[99, 100]
circMAP2K4	Tissues Cell lines	Upregulation	Proliferation (+)	circMAP2K4/hsa-miR-139-5p/YTHDF1	[93]

*Refs.* references; *(* +*)* promoting effects; *( −)* inhibiting effects

Most of circRNAs interact with miRNA as ceRNA and participate in the expression regulation of target genes by blocking the inhibitory effect of miRNA on their target mRNA, thus constructing circRNA-miRNA-mRNA regulation network in pathogenesis. Li et al. [68] reported that circPVT1 could up-regulate SIRT7 by binding with miR-3666, and SIRT7 could further adjust cell proliferation, cell cycle and transcription. Functional tests indicated that circPVT1 downregulation would reduce proliferation and increase cell apoptosis of HCC cell; similarly, hsa_circ_0056836 [69] might accelerate the progression of HCC via miR-766-3p/FOSL2 axis, in which FOSL2 was considered to be associated with photoperiodic regulation, fibrosis and even carcinoma; besides, circHIPK3, which is generated from the second exon of the HIPK3 gene, could promote cell proliferation and migration through AQP3, to transfer water and glycerol by the transmembrane channel, AQP3 played the important role in tumorigenesis and cancer progression by sponging of miR-124 [70, 71]; circ_0015756 [72] was proved highly expressed in HCC tissue, cell and even serum. Moreover, knockdown of circ_0015756 could inhibit tumor metastasis and invasion through miR-610/FGFR1; Yang et al. [73] reported that has_circ_0039053 could accelerate proliferation and invasion via the miR-637/USP21 axis in HCC cells; and Wu et al. [74] found circRASGRF2’s function as an oncogenic agent in HCC, and knockdown of circRASGRF2 suppressed the proliferation and migration of HCC cells by upregulating FAK expression through sponging miR-1224. The reported studies revealed that silence of circ_0091579 could restrain the progress of HCC via regulating miRNA, such as proliferation, migration and invasion in HCC cells [75].

**Fig. 4:**
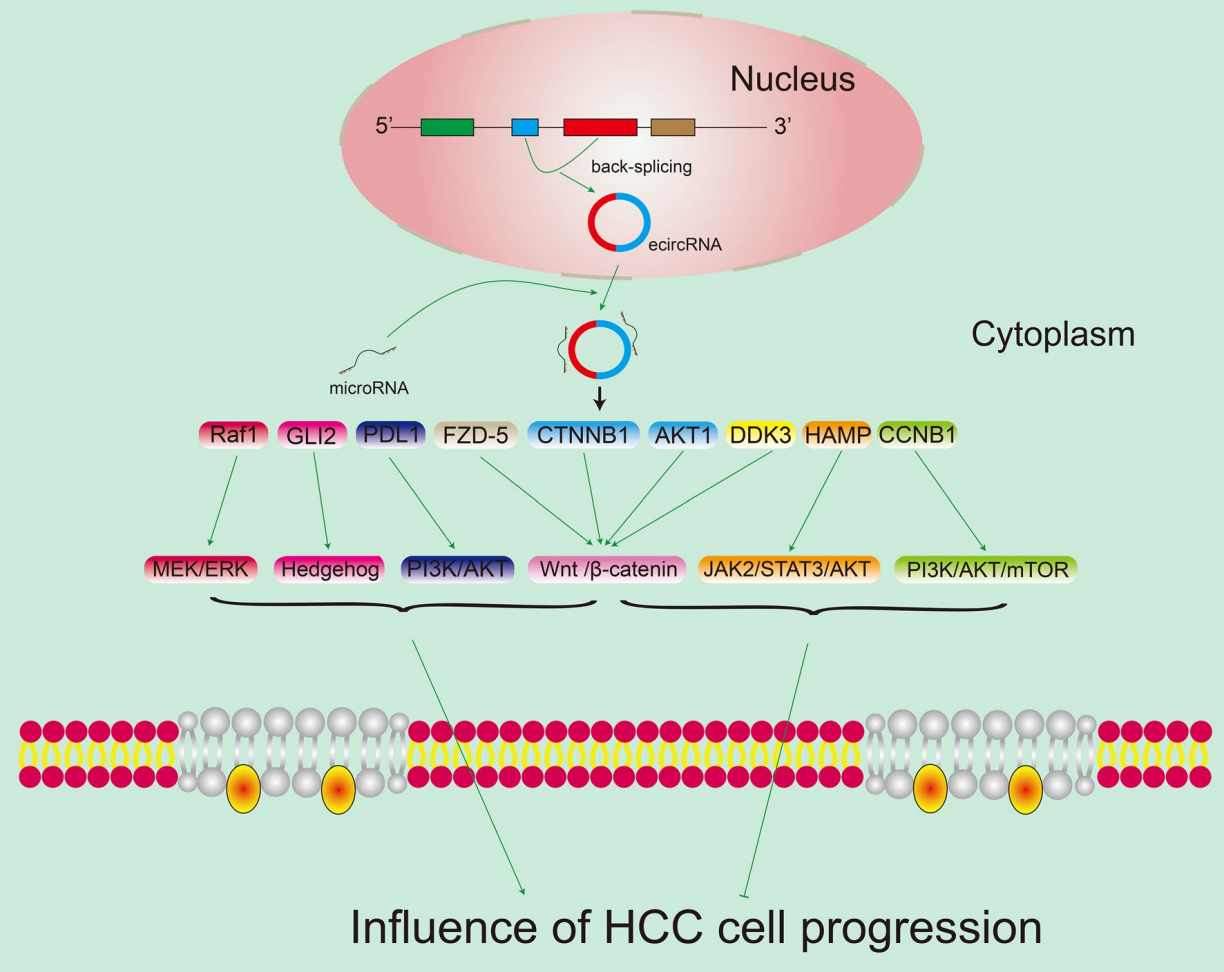
The signaling pathways of circRNA in HCC. The drawing mainly shows that circRNA mediated ceRNA network plays crucial roles in HCC progression through signaling pathways

CircRNA is also involved in the development of HCC through many signaling pathways exhibited in Fig. 4. Raf/MEK/ERK pathway plays an important role in cell growth, cell cycle and drug resistance, etc. It was verified that higher expression of circ_CDR1as could regulate MEK/ERK pathway via miR-1287/Raf1 axis, and then played positive roles in proliferation, migration, invasion, and EMT in HCC cells [76]. Similarly, hsa_circ_0003288 was overexpressed in HCC tissues and cells, which promoted the development of HCC by upregulating miR-145/PD-L1 axis via the PI3K/AKT signaling pathway [77]. It was found that circ_0067934 could activate signaling Wnt/β-catenin pathway by sponging of miR-1324 and improving FZD5 expression [78] and then promoting the proliferation, migration and invasion of HCC cells. CircZFR was proved to facilitate the progress of HCC through activating Wnt/β-catenin via regulating miR-3619-5p/CTNNB1 axis and miR-511/AKT1 signaling [79, 80]. It also reported that circZNF609 could accelerate the HCC development by activating the hedgehog pathway through regulation of miR-15a-5p/15b-5p and GLI2 expression. Knockdown of circZNF609 inhibited HCC cell proliferation, metastasis and stemness whereas boosted cell apoptosis [81].

The change of cell cycle is also a major factor affecting the progression of HCC. For instance, as members of the cell cycle family, CK6, p6 and p27 could influence cell proliferation by regulating the G1/S transition. High expression of circBACH1 [82] and circ-ZEB1.33 [83] could promote the proliferation of HCC cells via HuR/p27 and miR-200a-3p/CDK6, respectively. Besides, circ-ZEB1.33 could also be determined in serum and associated with tumor-node-metastasis (TNM) stages, which is important in the development stage of HCC.

CircRNA might also influence the occurrence and development of HCC through regulating downstream protein directly. Li et al. [84] found that overexpression of hsa_circ_0085616 could promote the proliferation, migration, and invasion of HCC cells by increasing of β-catenin, p-ERK, and p-AKT protein levels.

Epithelial to mesenchymal transition (EMT) refers to a biological process of epithelial cells transforming from differentiated properties to mesenchymal characteristics [85], it is a key part of cancer metastasis [86]. Twist1 was found could regulate expression of vimentin by circCul2, and then promote the EMT, tumor metastasis and malignancy of HCC [87].

Emerging studies showed angiogenesis played an important role in rapid growing and metastasis of tumors [88]. Yang et al. [89] reported that circCRIM1 was upregulated and could promote HCC proliferation and angiogenesis via miR-378a-3p/SKP2 axis. Li et al. [90] found that circ_0001955 regulated HCC proliferation, metastasis, angiogenesis, and apoptosis through targeting the FZD4 via sponging miR-646. It also suggested that CircGFRA1 was to be associated with proliferative, migration and angiogenic activity of HCC by binding miR-149 [91].

Recently, it reported that m^6^A RNA methylation modulators contributed to the progression and prognosis of HCC. CircKIAA1429 was overexpression in HCC tissues and cell lines, and it could promote HCC migration, invasion, and EMT with a mechanism of m^6^A-YTHDF3-Zeb1, due to YTHDF3 could improve the stability of Zeb1mRNA [92]. CircMAP2K4 could promote HCC cell proliferation through hsa-miR-139-5p/YTHDF1 axis. Hsa-miR-139-5p and YTHDF1 expression levels were associated with grade, stage and prognosis of HCC [93].

CircRNA is also related to clinical manifestations of HCC patients. For example, circPVT1 [94] and hsa_circ_102559 [95] were closely associated with the overall survival (OS), lymph node metastasis (LNM), and TNM stages. Upregulation of circ-Gprc5a [96], circHOMER1 [97], circFAT1 [98] and cirrc-CSPP1 [99, 100] were positively correlated with the enlargement of tumor size and the higher TNM stage as well as poorer prognosis.

To sum up the above-mentioned, circRNA plays an oncogenic agent role and facilitates development of HCC through different molecular regulatory mechanisms, which will contribute to the discovery of therapeutic targets for HCC in the future.

### CircRNA as suppressor in HCC

Compared with the high expression of circRNAs, some of them exhibit low expression which can inhibit the development of HCC and act as suppressors in HCC (Table 2).

**Table 2:** circRNA as suppressor in HCC

circRNA	Source of sample	Expression	Function	Mechanism/pathway	Refs.
circARSP91	Tissues Cell lines Animal	Downregulation	Growth (−)	AR/ADAR1/Cir ARSP91	[112]
circRNA7	Cell lines	Downregulation	Migration (−) Invasion (−) Vasculogenic mimicry (−)	AR/circRNA7/miRNA7-5p/VE-Cadherin/Notch4	[111]
circMTO1	Tissues Cell lines Animal	Downregulation	Proliferation (−) Invasion (−) Growth (−) Apoptosis (+)	circMTO1/miR9/p21	[101]
circRNA-5692	Tissues Cell lines Animal	Downregulation	Proliferation (−) Invasion (−) EMT (−) Growth (−) Apoptosis (+)	circRNA-5692/miR-328-5p/DAB2IP	[104]
circSMAD2	Tissues Cell lines	Downregulation	Migration (−) Invasion (−) EMT (−)	circSMAD2/miR-629	[103]
circADAMTS13	Tissues Cell lines	Downregulation	Proliferation (−) Apoptosis (+)	circADAMTS13/miR-484	[102]
hsa_circ_0079929	Tissues Cell lines Animal	Downregulation	Proliferation (−) Growth (−)	PI3K/AKT/mTOR	[108]
circ_0004913	Tissues Cell lines Animal	Downregulation	Proliferation (−) Migration (−) Invasion (−) Growth (−)	circ_000491/miR-184/HAMP/JAK2/STAT3/AKT	[109]
hsa_circ_0004018	Tissues Cell lines Animal	Downregulation	Proliferation (−) Migration (−) Growth (−) Apoptosis (+)	hsa_circ_0004018/miR-626/DKK3	[110]
hsa_circ_0005986	Tissues Cell lines	Downregulation	Proliferation (−)	hsa_circ_0005986/miR-129-5p/Notch1	[105]
circC3P1	Tissues Cell lines Animal	Downregulation	Proliferation (−) Migration (−) Invasion (−) Growth (−)	circC3P1/miR4641/PCK1	[106]
circSETD3	Tissues Cell lines Animal	Downregulation	Growth (−) Proliferation (−)	circSETD3/miR421/MAPK14	[107]

*Refs* references; *(* +*)* promoting effects; *( −)* inhibiting effects

CircRNA downregulation participates in proliferation, migration, invasion, and apoptosis by binding of miRNA, which has been proved was suppressor. For example, circMTO1 [101] acts as the sponge of oncogenic miR-9 to upregulate p21 expression and then suppresses progression of HCC. In another study, circADAMTS13 [102] was proved to inhibit HCC progression by sponging oncogenic miR-484. Moreover, downregulation of circSMAD2 [103], circRNA-5692 [104], hsa_circ_0005986 [105], circC3P1 [106] and circSETD3 [107] and circSETD3 were not only impeded the tumorigenesis of HCC but also correlated with clinicopathological characteristics. CircSMAD2 [103]was associated with differentiation of HCC tissues and circRNA-5692 [104] was correlated with AFP level, cirrhosis history, tumor size, and metastasis; hsa_circ_0005986 [105] was linked to chronic hepatitis B family history, tumor size, microvascular invasion (MVI) and BCLC stage; circC3P1 was negatively correlated with TNM stage, tumor size and MVI; circSETD was significantly associated with tumor size and differentiation of HCC tissues. All of the above-mentioned cases indicated the practical value to explore the therapeutic and prognostic targets of HCC.

Some circRNAs inhibit HCC development through some signaling pathways also exhibited in Fig. 4. For instance, hsa_circ_0079929 downregulation could inhibit the proliferation and cell cycle of HCC cells [108]. The functions of hsa_circ_0079929 were relevant to cell cycle regulatory protein CCNB1 and PI3K/AKT/mTOR signaling pathway. Wu et al. [109] found that circ_0004913 was partially inhibited the JAK2/STAT3/AKT signaling pathway through miR-184/HAMP axis, thus restraining the proliferation, metastasis, and glycolysis of HCC cells. Zhu et al. [110] reported that hsa_circ_0004018 suppressed proliferation and migration of HCC cells i*n-vivo* and inhibited wnt/β-catenin signaling pathway through interacting with miR-626/DKK3. Besides, overexpression of these three circRNAs could curb the growth of HCC in-vivo.

Besides, a recent study illustrated that androgen receptor (AR) could inhibit the formation of vasculogenic mimicry of HCC and influence tumor metastasis by AR-circRNA7/miRNA7-5p/VE-Cadherin/Notch4 signaling [111]. Liang et al. also found [112] that circARSP91, as one of the circRNAs suppressed by AR via upregulating ADAR1, could inhibit HCC tumor growth in vitro and vivo, and laid the foundation for exploring new HCC therapies by reactivation of this circRNA.

### CircRNA for potential diagnosis in HCC

Although cancer tissues can be detected with the size of less than 1 cm along with the development of imaging technologies such as magnetic resonance imaging and computerized tomography, it still represents a problem of a financial burden [113]. In a spite of the relatively low sensitivity and high rate of AFP (39.0–65.0%) for misdiagnosis of HCC, serumal AFP is still considered as the most commonly indicator for screening in the clinic [114]. Therefore, searching for new and reliable biomarkers is required and many types of circRNAs have been found as potential biomarkers in HCC (Table 3). The powerful multidimensional biomarkers can be efficiently used for prediction of patient response and disease diagnosis.

**Table 3:** circRNA as biomarker in HCC

Name of circRNAs	Sample	Expression	Function	Mechanism/pathway	Diagnosis/prognosis ability	Refs.
circZKSCAN1	Tissues Cell lines Animal	Downregulation	Growth (−) Migration (−) Invasion (−)	Function as a ceRNA	AUC (0.834) Sensitivity (82.2%) Specificity (72.4%)	[115]
circ-EPHB4	Tissues Cell lines Animal	Downregulation	Proliferation (−) Migration (−) Invasion (−) Apoptosis (+) Growth (−)	circ-EPHB4/HIF-1α/HIF-1α-PI3K-AKT and HIF-1α-ZEB1	AUC (0.861) Sensitivity (88.9%) Specificity (97.2%)	[118]
circ-TCF4.85	Tissues Cell lines Animal	Upregulation	Proliferation (+) Migration (+) Invasion (+) Growth (+) Apoptosis (−)	circ-TCF4.85/miR-486-5p/ABCF2	AUC (0.891) Sensitivity (86.8%) Specificity (87.0%)	[117]
hsa_circ_0003998	Tissues Cell lines Blood samples	Upregulation	–	Sponging miR-143-3p and PCBP1	AUC (0.894) Sensitivity (84.0%) Specificity (80.0%)	[53]
hsa_circ_0001445	Tissues Cell lines	Downregulation	Proliferation (−) Migration (−) Invasion (−) Apoptosis (+)	–	AUC (0.862) Sensitivity (94.2%) Specificity (71.2%)	[126]
circRNA 0068669	Tissues Cell lines	Downregulation	Associated with MVI and TNM stages	–	AUC (0.640) Sensitivity (59.0%) Specificity (71.0%)	[120]
has_circ_0005075	Tissues Cell lines	Upregulation	Proliferation (+) Migration (+) Invasion (+)	335miR-23b-5p, miR-93-3p, miR-581, miR-23a-5p	AUC (0.940) Sensitivity (83.3%) Specificity (90.0%)	[116]
hsa_circ_0016788	Tissues Cell lines Animal	Upregulation	Proliferation ( +) Invasion ( +) Apoptosis ( +) Growth (−)	hsa_circ_0016788/miR-486/CDK4	AUC (0.913) (95% CI 0.885–0.941)	[119]
circ-FOXP1	Tissues Cell lines Animal	Upregulation	Proliferation (+) Invasion (+) Apoptosis (−)	circ-FOXP1/miR-875-3p, miR-421/SOX9	AUC (0.9318) (95% CI 0.893–0.971)	[125]
circRNA_104075	Tissues Cell lines Animal	Upregulation	–	circRNA_104075/miR-582-3p/YAP	AUC (0.973) Sensitivity (96.0%) Specificity (98.3%)	[124]
circ-CDYL	Tissues Cell lines Animal	Upregulation	–	circ-CDYL/miR-892, miR-328-3p/HDGF and HIF1AN	AUC (0.640) (95% CI 0.550–0.720)	127
hsa_circ_0076251	Tissues	Downregulation	–	–	AUC (0.738) Sensitivity (71.3%) Specificity (64.0%)	[122]
hsa_circ_0028502	Tissues	Downregulation	–	–	AUC (0.675) Sensitivity (72.1%) Specificity (58.0%)	[122]

*AUC* area under the receiver operating characteristic curve; *CI* confidence interval; *EMT* epithelial to mesenchymal transition; *MVI* microvascular invasion; *TNM* tumor-node-metastasis

It was found that the area under the receiver operating characteristic curve (AUC) of circZKSCAN1 [115], has_circ_0005075 [116], hsa_circ_0016788 [52], hsa_circ_0003998 [53], circ-TCF4.85 [117] and circ-EPHB4 [118] reached above 0.800. Among them, has_circ_0005075 was related to tumor size of HCC; high expression of circ_0016788 [119] was correlated to high performance status score and large tumor size, increased Barcelona clinic liver cancer (BCLC) stage and abnormal AFP; the high expression of hsa_circ_0003998 [53] was associated with AFP, tumor size, differentiation and MVI and the lower expression of circ-EPHB4 [118] was positively associated with HBsAg and vascular tumor thrombus. All of them have great significance for diagnosing early HCC. There are also some circRNAs with lower AUC, whereas they are closely associated with the clinicopathological features of HCC. It was reported that hsa_circ_0068669 was associated with MVI and TNM stages and participated in metastasis of HCC, the AUC of curve of hsa_circ_0068699 was 0.64 with the values of sensitivity and specificity were 59.0% and 71.0% [120]. ZKSCAN1 mRNA and circZKSCAN1 are all the post-translational products from ZKSCAN1 gene. It was found that AUC of circZKSCAN1 was 0.834 and exhibited high sensitivity (82.2%) and specificity (72.4%), respectively, which was much higher than that of mRNA of ZKSCAN331 (AUC = 0.474) [115]. Therefore, it suggested that circZKSCAN1 demonstrated better efficiency in the diagnosis of HCC tissues than ZKSCAN1 mRNA.

Many circRNAs have been revealed unique features with the development of HCC, such as: the expression features of hsa_circ_0003570 [121], hsa_circ_0028502 and hsa_circ_0076251 [122] exhibiting stage-specific in HCC tissues, liver cirrhosis (LC) and chronic hepatitis (CH); and the expression levels are gradually increased. Moreover, hsa_circ_0003570 was closely related to tumor size, differentiation, MVI, BCLC stages and TMN stages; hsa_circ_0028502 were related to TNM stages, and the expression levels of hsa_circ_0076251 were related to BCLC stages and the presence of serum HbsAg, respectively, which suggested that these circRNAs also could be used as biomarkers at different stages in the development of HCC.

Serumal circRNA can also be employed as a non-invasive biomarker for the diagnosis of HCC. It showed that the ROC curve analysis for hsa_cirR_00156, hsa_cirR_000224 and hsa_cirR_000520 [123] were employed for discrimination between HCC patients and the healthy group, the AUC were 0.839, 0.974 and 0.943, respectively, which were higher than that of AFP (AUC = 0.726). Moreover, combining these three circRNAs displayed a sensitivity of 100% and a specificity of 83.3%. In another study, the level of circ_104075 [124] in serum was positively related to the stage of HCC. In addition, circ_104075 might be a potential diagnostic biomarker with an AUC of 0.973, the sensitivity and specificity values were 96.0% and 98.3%, respectively, which was also higher than that of AFP for predication of HCC. Moreover, the m6A modification could enhance the interaction between miR-582-3p and YAP3′UTR, and contributed to circRNA_104075 promoted HCC progression by miR-582-3p/YAP. It also displayed that serumal circ-FOXP1 plays an important role in the diagnosis and metastasis of HCC with the AUC of 0.932 and was associated with the tumor size, MVI and TNM stage [125].

Zhang et al. [126] found that the expression level of plasma hsa_circ_0001445 in HCC patients, cirrhosis patients and hepatitis B patients were lower than those in healthy group. Moreover, ROC curve was used to distinguish HCC patients from the healthy control (AUC = 0.862), cirrhosis (AUC = 0.672) and the hepatitis B patients (AUC = 0.764) according to the plasma level of hsa_circ_0001445. Besides, the combination of hsa_circ_0001445 and AFP revealed better diagnostic value than any of them. Another study [127] demonstrated that circ-CDYL combining with HDGF and HIF1AN were proved as promising biomarkers for diagnostic of early HCC than that of AFP with the odds ratios (ORs) of 124.58 (95% CI 13.260–1170.560).

### CircRNA as potential prognostic biomarker in HCC

HCC is characterized as high degree of malignancy, poor prognosis, low 5-year survival rate and high recurrence rate. At present, AFP and routine liver function tests are often used as monitoring methods to predict the recurrence of HCC. In addition, α‐fetoprotein‐L3 and Golgi protein73 play important role in prediction of HCC [128]. However, they performed lower sensitivity and limited efficiency. It is showed that circRNA is closely associated with the recurrence-free survival (RFS) and OS of HCC by univariate and multivariate Cox regression analysis, dysregulated circRNA might be used as a prognostic factor for the selection of rational treatment regimens for HCC, due to circRNA shows stable conserved and high specificity in different tissues even the stages of disease [129]. For instance, hsa_circ_0128298 [130], circLARP4 [131], circTP63 [132], circBIRC6 [133], hsa_circ_104348 [134] and circZNF566 [135], etc., were overexpressed in HCC tissues and cell lines. Kaplan–Meier survival analysis showed that HCC patients with circRNA high expression and had shorter overall survival than those with low expression. In addition, hsa_circ_0128298 [130], circLARP4 [131] and circBIRC6 [133] were testified to be the independent prognostic factors of OS by multivariate Cox regression analysis. Moreover, studies have found that high expression of hsa_circ_0128298 was associated with vascular cancer embolus, LNM and organ metastasis; overexpression of circTP63 [132] was related to TNM stage, tumor differentiation, and LNM; circLARP4 [131] and circZNF566 [135] were associated with clinicopathological features, including tumor differentiation, tumor size and TNM stage, etc., circ-BIRC6 [133] was associated with TNM stage and MVI in HCC tissues; hsa_circ_104348 [134] expression level was related to tumor size, LNM as well as TNM. Besides, univariate and multivariate Cox regression analysis showed circZKSCAN1 was an independent prognostic factor of OS and RFS by binding FMRP, and the combination of circZKSCAN1 and CCAR1 could improve the prognostic value of HCC [136].

Moreover, cSMARCA5 [137], hsa_circ_0001649 [138] and circTRIM33-12 [139] were downregulated in HCC tissues. It reported that cSMARCA5 was associated with tumor differentiation, tumor stage, tumor size and MVI; and circTRIM33-12 was linked to multiple tumors, tumor size, encapsulation invasion, AFP levels and MVI. Furtherly, hsa_circ_0001649 was related to OS, the lower expressions of cSMARCA5 and circTRIM33-12 in HCC patients exhibited poor OS and RFS by Kaplan–Meier analysis; and multivariate analyses indicated that both them were the independent risk factors of OS and RFS. Therefore, they revealed the critical guiding significance to the prognosis of HCC. Recently, circRNAs act as potential prognosis biomarkers were listed in Table 4.

**Table 4:** circRNA as potential prognosis biomarker

Name of circRNAs	Sample	Expression	Prognosis	Kaplan–Meier(P)	Univariate analysis (P)	Multivariate analysis (P)	Mechanism	Refs.
circ-0128298	Tissues Cell lines	Upregulation	OS	0.003	0.009	0.014	–	[130]
circLARP4	Tissues Cell lines Animal	Downregulation	OS	–	–	0.001	circLARP4/miR-761/RUNX3/p53, p21	[131]
			RFS	–	–	0.024		
circ-BIRC6	Tissues Cell lines Animal	Upregulation	OS	< 0.050	0.009	0.014	circ-BIRC6/miR-3918/Bcl2	[133]
hsa_circ_104348	Tissues Cell lines	Upregulation	OS	0.0041	–	–	hsa_circ_104348/miR-187-3p/RTKN2	[134]
cSMARCA5	Tissues Animal cell lines	Downregulate	OS	0.0004	–	0.001	DHX9/cSMARCA5/miR-17-3p, miR-181b-5p/TIMP3	[137]
			RFS	0.008	–	0.021		
circZKSCAN1	Tissues Cell lines Animal	Upregulation	OS	< 0.001	< 0.001	< 0.001	QKI5/circZKSCAN/FMRP/CCAR1	[136]
			RFS	< 0.001	< 0.001	< 0.001		
circTP63	Tissues Cell lines Animal	Upregulation	OS	0.0169	–	–	circTP63/miR-155-5p/ZBTB18	[132]
circZNF566	Tissues Cell lines Animal	Upregulation	OS	0.018	–	–	circZNF566/miR-4738-3p/TDO2	[135]
			DFS	0.007	–	–		
hsa_circ_0001649	Tissues Cell lines	Downregulation	OS	0.007	0.015	0.011	–	[138]
circTRIM33–12	Tissues Cell lines Animal	Downregulation	OS	0.0007	0.001	0.007	circTRIM33–12/miR-191/TET1	[139]
			RFS	–	0.001	0.005		

*Refs* references; *OS* overall survival; *RFS* recurrence-free survival

## CircRNA of HBV related HCC

HBV infection is a major risk factor in the high incidence of HCC in the areas of Asia and sub‑Saharan Africa [140]. It reported that high expression of circRNA_100338 in HCC tissues compared with paired pericancerous live tissues samples with hepatitis B, the cumulative survival rate (72.0%) of HCC patients in the circRNA_100338-high group was significantly lower than that of the low expression group (42.9%); circRNA_100338 was highly correlated with TNM stage, invasion and metastasis in HCC via antagonizing miR-141-3p [141]. Hsa_circ_0027089, a plasmatic circRNA, was proved to discriminate HBV-related HCC from HBV-related cirrhosis and healthy participants with AUC values of 0.765 and 0.794 [142]. It might act as a potential biomarker for clinical diagnosis and evaluation in HCC with HBV. Another study identified 24 and 23 circRNAs with up-regulation and down-regulation by microarray in three paired HBV‑related HCC tissues and adjacent non‑tumorous tissues; and the differentially expressed circRNA/miRNA interactions were predicted by miRNA target prediction software [143]. Here it can be concluded that circRNA plays a critical role in distinguishing different types of HCC, e.g., plasmatic circRNA could act as a valuable diagnosis biomarker to distinguish HBV-related HCC and HBV-related cirrhosis.

### CircRNA in other liver diseases

#### Chronic hepatitis B (CHB)

Zhou et al. [144] provided the first evidence for differentially expressed circRNAs in CHB. Interestingly, a bioinformatics method for identification of CHB-associated circRNA and the silico analysis were established for four predicted circRNA-miRNA-mRNA pathways in progression of HBV-associated liver disease. They found 72 up-regulated and 95 down-regulated circRNAs were changed more than twice in CHB tissues compared with the normal tissues. Subsequently, four pathways of circRNA-miRNA-mRNA were also discovered, including: hsa_circ_0005389-miR-4505/miR-6752-5p/miR-5787-IRF7, hsa_circ_0000650-miR-6873-3p-TGFβ2, hsa_circ_0000650-miR-210-5p-HBV and hsa_circ_0000038-miR-370/miR-939-HBV. Besides, it was found that circ_0004812 was upregulation in CHB and promoted FSTL1 expression by binding to miR-1287-5p [145]. Additionally, the enhanced expression mRNA and its related protein of IFN-α and IFN-β in the circ_0004812 knockdown cells, suggests that circ_0004812 could regulate HBV-induced immune suppression by miR-1287-5p/FSTL1 axis. They demonstrated a new perspective on the new pathogenesis of CHB through circRNA.

#### Hepatic steatosis

Hepatic steatosis is recognized as one of the common chronic liver diseases in western countries and the Asia–Pacific area [146–148]. A large proportion of patients with non-alcoholic fatty liver disease (NAFLD) may further develop non-alcoholic steatohepatitis (NASH), fibrosis, cirrhosis, or even HCC [149]. Some circRNAs derived from mitochondria were down-regulated in NASH [150]. Among them, hsa_circ_0089762 was associated with mitochondrial reactive oxygen species (mROS) release and metaflammation in NASH. In terms of the mechanism, hsa_circ_0089762 directly interacts with ATP5B to block the binding of ATP5B to CypD and inhibit the opening of mitochondrial permeability transition pore, thus inhibiting mROS output and fibroblast activation. Therefore, mitochondrial circRNA could be a new therapeutic target for NASH. circRNA_002581 overexpression was found to up-regulate CPEB1 by sponging miR-122 in the NASH mice model [151]. Further study indicated that circRNA_002581-miR-122-CPEB1 was linked to autophagy by PTEN-AMPK-mTOR pathway. Besides, circRNA_0046367 and circRNA_0046366 could act as the antagonists of miR-34a are involved in NAFLD [152, 153]. Therefore, these pathways provide novel approaches for NASH pathogenesis, diagnosis and treatment.

#### Hepatoblastoma

Hepatoblastoma is the most common malignant liver cancer in infants and toddlers [154]. Circ_0015756 was found significantly up-regulated in human hepatoblastoma tissues and cell lines and circ_0015756 siRNA decreased the viability, proliferation and invasion ability of hepatoblastoma cells in vitro by sponging miR-1250-3p [155]. Li et al. [156] demonstrated that circSETD3 downregulated in Hepatoblastoma tissues and cell lines, and it exerted as a tumor suppressor to inhibit proliferation, migration, EMT process, and induce apoptosis by miR-423-3p/Bim axis.

## Conclusions

By these tokens, a large number studies have shown that circRNAs are abnormally expressed in liver diseases and play a vital role in regulating different molecules, signaling pathways, pathophysiological activities, etc. However, the study on the relationship between circRNA and liver disease is still in the stage of infancy. Most of the molecular mechanisms of circRNA in liver diseases are remained unclear, although the development and clarifying researches of circRNA in liver diseases are accelerating. Besides, there are still many deficiencies in the study of circRNA. Firstly, compelling evidences are mainly focused on HCC, while a few are concentrated on other liver diseases. Secondly, the sample sizes in many studies of circRNA as diagnostic biomarker are relatively small, and methodologies are required to be standardized to eliminate possible artifacts. Last but not least, most circRNAs are derived from the pathological tissues of the liver, the role and function of circRNA in serum, exosomes and microvesicles are still seldom reported. We believed that the application of circRNA has a broad prospect in the drug therapeutic target, diagnosis and treatment of liver diseases with the improvement of detection, validation and functional analysis methods of circRNA.

## Data Availability

Data will be provided based on reasonable request.

## Abbreviations

AFP: Alpha fetoprotein
AR: Androgen receptor
AUC: Area under the receiver operating characteristic curve
BCLC: Barcelona clinic liver cancer
CH: Chronic hepatitis
ciRNA: Circular intronic RNA
circRNA: Circular RNA
dd PCR: Droplet digital PCR
DECs: Differential expression of circRNAs
EIciRNA: Exon–intron circRNA
EcircRNA: Exonic circRNAs
HCC: Hepatocellular carcinoma
HuR: Human antigen R
LC: Liver cirrhosis
LNM: Lymph node metastasis
m^6^A: N6-methyladenosine
mROS: Mitochondrial reactive oxygen species
MVI: Microvascular invasion
NASH: Non-alcoholic steatohepatitis
ncRNA: Non-coding RNA
NGS: Next generation sequencing
ORs: Odds ratios
OS: Overall survival
RFS: Recurrence-free survival
RNA-seq: RNA sequencing
TNM: Tumor-node-metastasis
